# PDCD1 as a targetable immune checkpoint hub: therapeutic insights for ibrutinib-resistant CLL management

**DOI:** 10.1007/s10238-025-01942-2

**Published:** 2025-11-18

**Authors:** Niloufar Sadat Kalaki, Elham Karimi, Kasra Allaei Rouzbahani, Mozhgan Ahmadzadeh, Seyed Mohammad Akrami

**Affiliations:** 1 Student Research Committee, School of Medicine, Shahid Beheshti University of Medical Sciences, Tehran, Iran; 2 Department of Medical Genetics, Shahid Beheshti University of Medical Sciences, Tehran, Iran; 3 Department of Medical Genetics, School of Medicine, Tehran University of Medical Sciences, Poursina St., P.O. Box: 14176-13151, Tehran, Iran; 4 Department of Medical Genetics, Faculty of Medicine, Hormozgan University of Medical Sciences, BandarAbbas, Iran; 5 Student Research Committee, Faculty of Medicine, Hormozgan University of Medical Sciences, Bandar Abbas, Iran; 6 Department of Cellular and Molecular Biology, Faculty of Biological Sciences, Kharazmi University, Tehran, Iran

**Keywords:** Chronic lymphocytic leukemia (CLL), Ibrutinib resistance, PPI network, Immune checkpoint

## Abstract

Chronic lymphocytic leukemia (CLL) is a type of cancer that affects the blood and bone marrow, specifically involving the overproduction of abnormal lymphocytes. Using two independent datasets (GSE249956 and GSE98206), differentially expressed genes (DEGs) were identified between ibrutinib-resistant and sensitive CLL samples. Protein–protein interaction (PPI) network analysis revealed key hub genes related to resistance. Three common hub genes were shared across datasets: PDCD1, CD1C, and ITGB2. PDCD1, encoding the immune checkpoint protein PD-1, was selected for deeper investigation due to its important role in immune regulation and relevance in drug resistance. Post-transcriptional regulation of PDCD1 was explored by the construction of competing endogenous RNA (ceRNA) networks linking lncRNAs and miRNAs that may modulate PDCD1 expression, indicating complex upstream regulatory mechanisms. Transcription factors potentially regulating PDCD1 were identified, suggesting layers of transcriptional control contributing to ibrutinib resistance. Drug-gene interaction analysis showed that although PDCD1 is not directly targeted by ibrutinib. This implicates immune checkpoint blockade as a promising therapeutic strategy to overcome or complement resistance in CLL. In the broader context, PD-1 expression in CLL cells is linked to active proliferation and immune escape. Overall, our findings emphasize PDCD1’s central role in ibrutinib resistance through immune checkpoint pathways and support the rationale for combining BTK inhibitors with immune checkpoint blockade therapies in resistant CLL cases.

**Supplementary Information:**

The online version contains supplementary material available at 10.1007/s10238-025-01942-2.

## Introduction

Chronic lymphocytic leukemia (CLL) is the most common type of leukemia in adults, with a median age of 72 years in Western countries, characterized by the progressive accumulation and malignancy of mature B cells in the blood, bone marrow, and secondary lymphoid tissues ultimately leading to lymphocytosis, bone marrow infiltration, lymphadenopathy, and splenomegaly. Because the disease often affects older adults, approximately 95% of patients have at least one underlying disease [1]. A large percentage of patients with CLL are asymptomatic at the time of diagnosis, and 30% of them do not require treatment for CLL [2]. Today, with the increasing development of science, our understanding of cancer and CLL, as well as diagnostic and therapeutic approaches, has made great progress. Therapeutic approaches such as chemotherapy, despite their success in achieving remission in CLL patients, are associated with limitations such as bone marrow suppression and infection. In recent decades, the treatment of CLL has become more targeted [3]. One of these drugs that has revolutionized the treatment of chronic lymphocytic leukemia (CLL) is an oral Bruton tyrosine kinase (BTK) inhibitor called ibrutinib. This drug, which has become a standard treatment for CLL, is approved as a first-line treatment for relapsed and refractory CLL [4].

However, resistance to covalent BTK inhibitors like ibrutinib can develop, often due to mutations in the BTK gene. For patients with covalent BTK inhibitor-resistant CLL, current clinical guidelines (e.g., ESMO and NCCN) recommend venetoclax-based regimens, typically combined with anti-CD20 monoclonal antibodies such as rituximab or obinutuzumab, and non-covalent BTK inhibitors like pirtobrutinib, which have shown efficacy in overcoming resistance [5, 6]. In addition to directly inhibiting BTK, ibrutinib also modulates the immune system and alters the CLL microenvironment by blocking multiple signaling pathways and inducing cell apoptosis [7]. To restore T-cell activity and prevent cancer cells from evading the immune system, several immune checkpoint inhibitors, including Nivolumab, Pembrolizumab, Dostarlimab, and Durvalumab, have been investigated for their potential in treating CLL by targeting the PD-1/PD-L1 pathway [8]. However, because clinical trials have not yet shown consistent or meaningful clinical advantages in this patient population, major clinical recommendations (e.g., ESMO and NCCN) do not currently suggest these medicines for the treatment of CLL, despite encouraging preclinical and early clinical results. For instance, pembrolizumab inhibits the T-cell pathway, and because T-cell function is compromised in CLL patients, cells in the tumor microenvironment express the PDCD1 gene more frequently [8–10]. Programmed cell death protein 1 (PDCD1) is an immunosuppressive receptor expressed on activated T-cells whose ligand is on tumor cells, such as CLL cells. Studies have shown that the interaction of PDCD1 and its ligand leads to impaired T-cell function and immune evasion by cancer cells; therefore, targeting PDCD1 and its ligand with inhibitory drugs leads to the restoration of T-cell immune function and antitumor activity [11]. Another study that has led to significant advancements in the early detection, effective treatment, or development of cancer is the discovery of non-coding RNAs (ncRNAs) and microRNAs (miRNAs). These transcripts, which range from a few base pairs to several kilobases and are involved in the regulation of gene networks and intracellular pathways, other transcripts, or proteins, have also played an important role in chronic lymphocytic leukemia (CLL) as potential biomarkers and therapeutic targets [12]. LncRNAs such as MALAT1, XIST, and NEAT are increased in CLL, leading to increased drug resistance in tumor cells and immune evasion [13]. MicroRNAs may also play an important role in CLL pathology. Research has shown that microRNAs regulate gene expression, and any disruption in their normal function can alter the expression levels of genes involved in tumor development/progression. Hence, these transcripts have the potential to be used as diagnostic and therapeutic biomarkers for CLL [14]. This study aims to investigate PDCD1 as a central gene in CLL and the drug resistance caused by microRNAs and LncRNAs that target it. It is hoped that this study will identify PDCD1 as a gene with predictive value, thereby improving the diagnosis and effective treatment of CLL patients.

## Methods and materials

### Microarray data acquisition and preprocessing

The microarray dataset GSE249956 was downloaded from the Gene Expression Omnibus (GEO) database (https://www.ncbi.nlm.nih.gov/geo/) [15]. The GPL10558 platform (Illumina HumanHT-12 V4.0 expression beadchip) served as the basis for this dataset, which comprises gene expression profiles of Ibrutinib-treated chronic lymphocytic leukemia (CLL) patients who were classified as either Ibrutinib-sensitive or Ibrutinib-resistant.

The GEO2R online tool was used to process the raw expression data. This tool compares two or more groups of samples within a GEO series. Given that hundreds of thousands of microarray gene expression datasets are freely accessible for download and use, GEO may prove to be a valuable resource. The following conditions are satisfied by the four datasets: Ibrutinib-treated human CLL samples and a case–control group were both present in this trial, and there were at least 40 samples. The data that were chosen as DEGs and used for network design were those with adjusted *P*-values < 0.05, logFC (fold change) ≥ 1.5, and logFC (fold change) ≤ − 1.5.

### Validation dataset analysis

Analysis of a second dataset, GSE98206, was done to verify the DEGs derived from GSE249956 [15]. Gene expression data from CLL patients both before and after Ibrutinib treatment are included in this dataset, which also makes use of the GPL10558 platform. The pre-treatment and post-treatment groups’ differential expression was analyzed using the GEO2R program. DEGs sensitive to Ibrutinib treatment were identified using the same thresholds: adjusted *p*-value < 0.05, logFC (fold change) ≥ 1, and logFC (fold change) ≤ − 1.

### Identification of common DEGs between datasets

An online Venn diagram tool (http://bioinformatics.psb.ugent.be/webtools/Venn/) was used to compare the lists of DEGs from GSE249956 and GSE98206 in order to find genes that might be implicated in Ibrutinib resistance. DEGs that overlapped were chosen for additional examination because they were thought to be strong candidates linked to the Ibrutinib response.

### PPI network construction and performance analysis

DEGs were imported into the STRING server (https://string-db.org; version 11.5) in order to identify the hub genes according to their protein–protein interaction (PPI) network. The centrality parameters for the PPI network were determined using a PPI network with centrality factors including degree, betweenness, and proximity. The output file produced by STRING was imported into Cytoscape as a source for the study of important genes in the network, since Cytoscape (version 3.6.0) was used as a tool to build the PPI network. Cytoscape was used to identify hub genes based on centrality criteria such as degree, betweenness, and closeness. These genes can be used as candidates to identify hub genes.

### Selection of key overlapping gene and drug interaction analysis

A crucial overlapping gene was chosen due to its significance in the PPI network and its established role in immune signaling and drug response. This gene was subsequently analyzed using the DrugBank database to discover known or anticipated interactions with Ibrutinib or various other anti-cancer medications [16]. A literature review was also conducted to assess the gene's role in Ibrutinib sensitivity or resistance.

### Expression and clinical relevance analysis

The expression levels and frequency of alterations for the chosen gene in CLL were evaluated using cBioPortal for Cancer Genomics (https://www.cbioportal.org/) [17]. The clinical significance, including its connection to patient survival, was evaluated whenever possible. This process aided in confirming the gene’s functional role concerning CLL advancement and resistance to treatment.

### Construction of the lncRNA-miRNA-mRNA interaction network

A competitive endogenous RNA (ceRNA) network was built in order to examine the putative post-transcriptional regulation mechanisms of the chosen hub gene. The ceRNA network was then formed by combining the interactions among lncRNA, miRNA, and mRNA, and Cytoscape (version 3.6.0) was utilized for visualization [18]. This network provides insight into the competitive binding relationships between lncRNAs and mRNAs through shared miRNAs, and how such regulation may influence drug resistance mechanisms in chronic lymphocytic leukemia (CLL).

### Construction of transcription factor regulatory network

Transcription factors (TFs) that might control the expression of the chosen hub gene were identified by retrieving TF gene interaction data from public databases, such as TRRUST (https://www.grnpedia.org/trrust/) [19]. Transcription factors (TFs) with substantial evidence of binding or regulatory impact on the hub gene were chosen. The regulatory network linking TFs and the hub gene was built and visualized with Cytoscape (version 3.6.0), allowing for the identification of crucial TFs that could influence gene expression and play a role in drug resistance mechanisms in chronic lymphocytic leukemia.

### Statistical analysis

We calculated the values of the DEGs sourced from the GEO DataSets. Adjusted *P*-values below 0.05 were deemed statistically significant and were included as significant data. For GO and KEGG enrichment analyses, a *P*-value of less than 0.05 is regarded as the threshold for significant results. In the Analysis-Box Plots module of GEPIA, using settings of *p*-values < 0.05, log 2FC < 1, and matching TCGA Normal to GTEx Data, we can investigate the expression levels of genes linked to CLL.

## Results

### Identification of differentially expressed genes (DEGs) in ibrutinib-resistant CLL

**Fig. 1:**
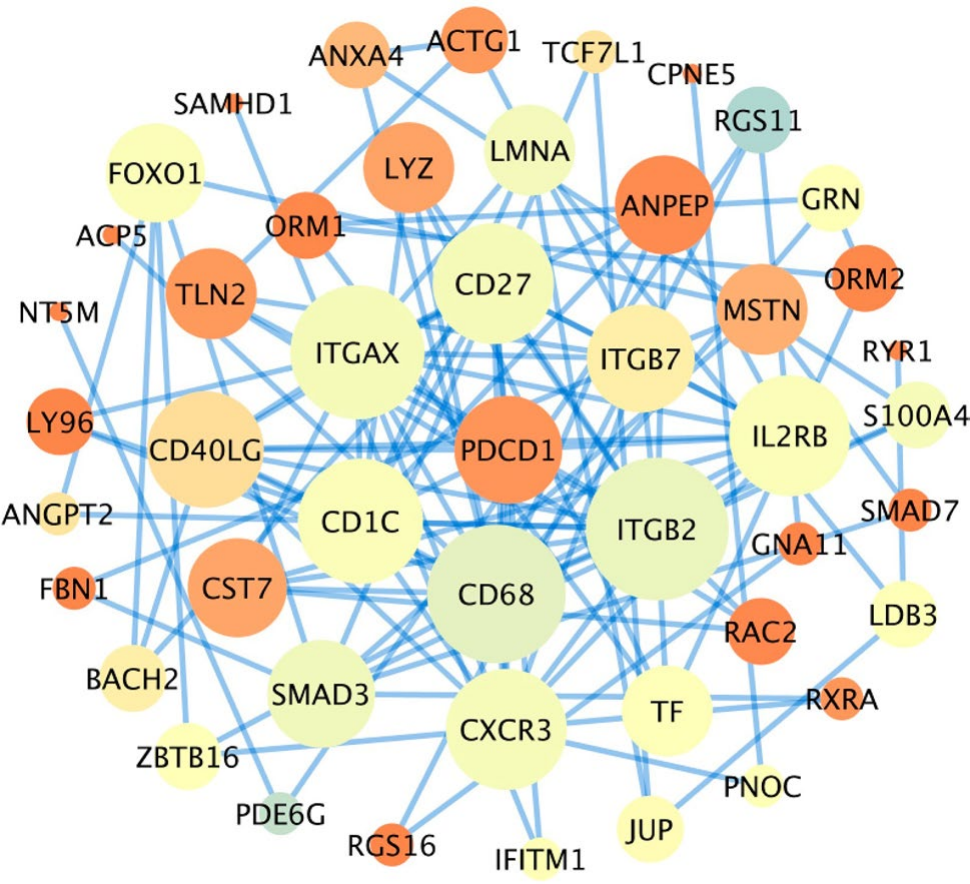
Protein–Protein Interaction (PPI) analysis. The size and the color of nodes represent the degree and betweenness, respectively. PPI network of Hub genes of the commonly differentially expressed genes from the GSE249956

Microarray data from GSE249956 were examined to find differentially expressed genes (DEGs) between samples of CLL that are resistant to ibrutinib and those that are sensitive. Applying the criteria of logFC ≥ 1.5 and logFC ≤ − 1.5 along with an adjusted *p*-value of less than 0.05, we identified a total of 132 genes that were upregulated and 200 genes that were downregulated. The leading DEGs common across both datasets underwent protein–protein interaction (PPI) network analysis using the STRING database and were visualized with Cytoscape (Fig. 1). We identified 47 overlapping hub genes. (number of nodes: 47, clustering coefficient: 0.404, network centralization: 0.238) (Supplementary Table 2).

### Identification and validation of hub genes through cross-dataset comparison

**Fig. 2:**
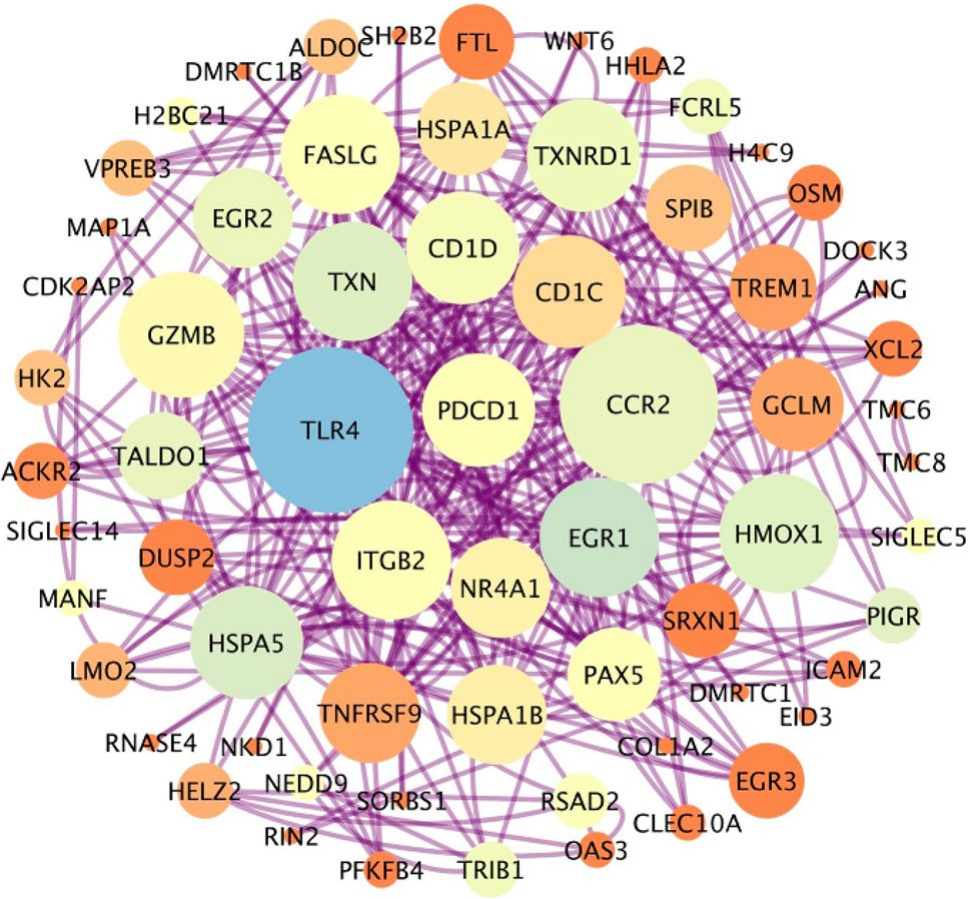
Protein–Protein Interaction (PPI) analysis. The size and the color of nodes represent the degree and betweenness, respectively. PPI network of Hub genes of the commonly differentially expressed genes from the GSE98206

To determine key genes linked to resistance, differentially expressed genes (DEGs) from GSE249956 were analyzed alongside those from an alternative dataset, GSE98206, with criteria set at logFC ≥ 1 and logFC ≤ − 1, along with an adjusted *p*-value < 0.05. The genes that were found to be shared were regarded as high-confidence candidates. A total of 97 genes were found to be upregulated and 32 downregulated. The most significant DEGs present in both datasets were analyzed for protein–protein interactions (PPI) using the STRING database, and the results were visualized with Cytoscape (Fig. 2). A total of 66 hub genes were identified through this overlap. (number of nodes: 66, clustering coefficient: 0.372, network centralization: 0.200) (Supplementary Table 2).

### Identification of common hub genes across datasets

**Fig. 3:**
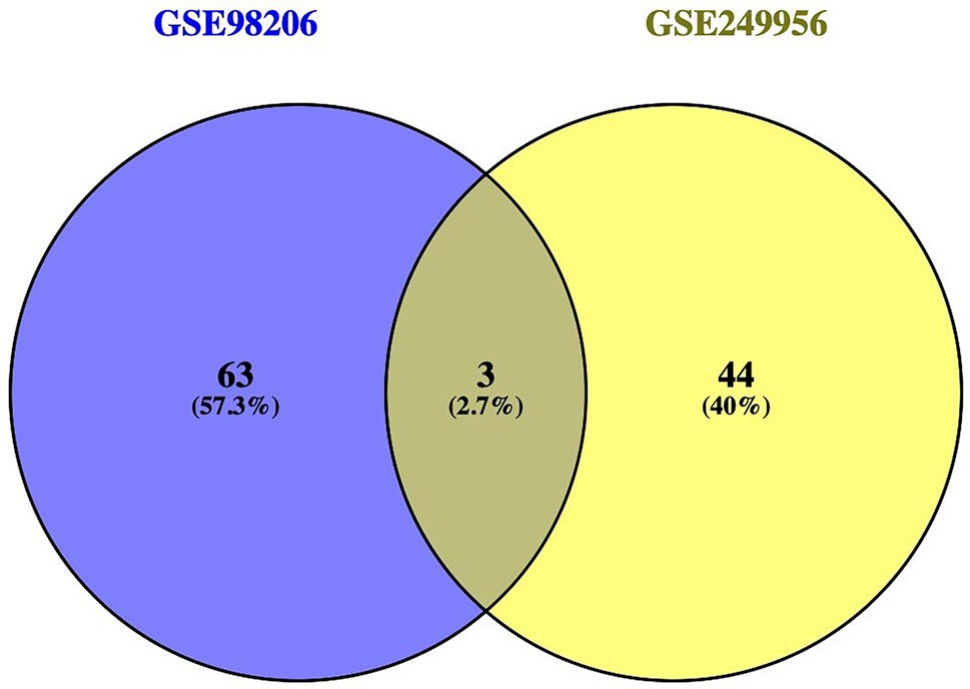
Venn diagram of overlapping hub genes between GSE249956 and GSE98206

To improve the accuracy of hub gene identification, the differentially expressed genes (DEGs) from GSE249956 and GSE98206 were analyzed separately using STRING and Cytoscape. Hub genes were determined based on degree centrality with the help of the cytoHubba plugin in both datasets. After constructing protein–protein interaction (PPI) networks and extracting hub genes from each dataset, a comparison was made to pinpoint overlapping hub genes. The analysis uncovered three common hub genes found in both datasets: PDCD1 (Programmed Cell Death Protein 1), CD1C (CD1c Molecule), and ITGB2 (Integrin Subunit Beta 2). Among these, PDCD1 was chosen for further investigation due to its significant role in immune checkpoint regulation and its growing importance in drug resistance pathways, especially in the context of ibrutinib-treated chronic lymphocytic leukemia (CLL). A Venn diagram was created to illustrate the overlapping hub genes (Fig. 3).

### The ceRNA regulatory network of PDCD1 was established to investigate its potential post-transcriptional regulation

miRNAs predicted to target PDCD1 were sourced from miRTarBase, TargetScan, and miRWalk. The overlapping miRNAs were subsequently utilized to pinpoint possible lncRNAs that interact with them through starBase v3.0.

### Identification of transcription factors influencing PDCD1

Transcription factors that modulate PDCD1 were pinpointed through the TRRUST database. Numerous TFs with significant evidence of interaction with PDCD1 were found, including [TF1, TF2, TF3].

### Analysis of drug–gene interactions and their therapeutic consequences

To investigate the possible therapeutic outcomes of the identified hub gene PDCD1, an analysis of drug–gene interactions was conducted using the DrugBank and DGIdb databases. Although PDCD1 is not a recognized direct target of ibrutinib, several immune checkpoint inhibitors were discovered to directly interact with PDCD1.

In particular, four drugs—Nivolumab, Pembrolizumab, Dostarlimab, and Durvalumab—were identified as targeting PDCD1 or its associated pathway. These medications work by inhibiting the PD-1/PD-L1 interaction, thus reactivating T-cell-mediated immune responses. All four drugs are approved by the FDA for use in various cancer types and have well-defined mechanisms for immune modulation (Table 1).

**Table 1:** Detailed Drug–Gene interactions for PDCD1 Identified via DrugBank and DGIdb

Drug name	Drug type	Mechanism of action	Source	Therapeutic status	FDA approval	Approved indications
Nivolumab	Immune checkpoint inhibitor	Fully human IgG4 anti–PD-1 antibody; Blocks PD-1/PD-L1 interaction, restoring T cell function	DrugBank	Approved	Yes	Melanoma, NSCLC, Hodgkin lymphoma, RCC, etc
Pembrolizumab	Immune checkpoint inhibitor	Humanized IgG4 anti–PD-1 antibody; Inhibits PD-1 to enhance immune response	DrugBank	Approved	Yes	Melanoma, NSCLC, HNSCC, urothelial carcinoma, etc
Dostarlimab	Anti–PD-1 monoclonal antibody	Humanized IgG4 antibody blocks PD-1/PD-L1 to restore immune surveillance	DGIdb	Approved	Yes	dMMR/MSI-H endometrial cancer, other solid tumors
Durvalumab	Anti–PD-L1 monoclonal antibody	Fully human IgG1 antibody targeting PD-L1; Blocks PD-L1 interaction with PD-1 and CD80	DGIdb	Approved	Yes	NSCLC, urothelial carcinoma, SCLC, etc

**Fig. 4:**
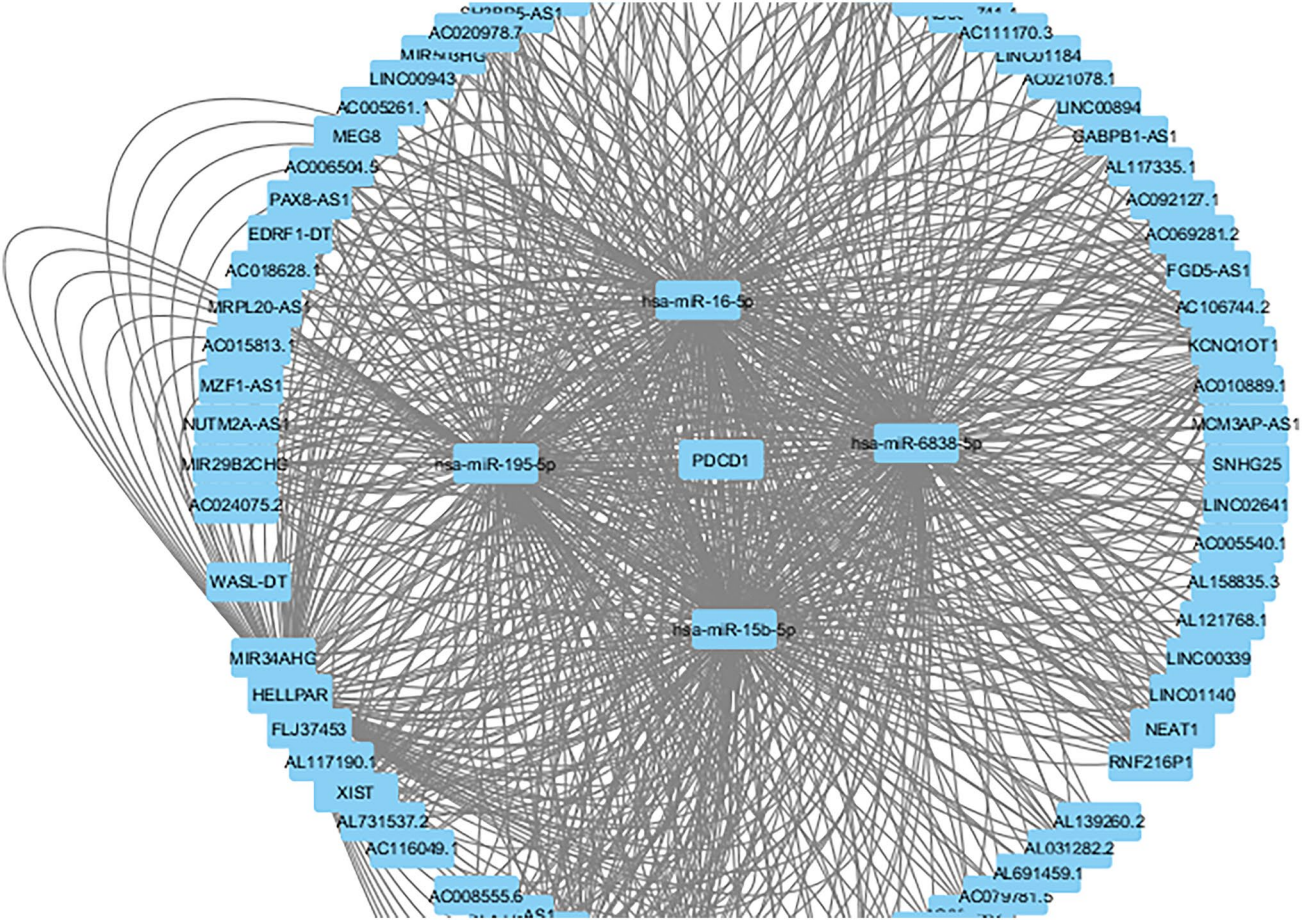
The final lncRNA–miRNA–PDCD1 network was visualized using Cytoscape, indicating potential upstream non-coding RNA regulators that may modulate PDCD1 expression in CLL

**Fig. 5:**
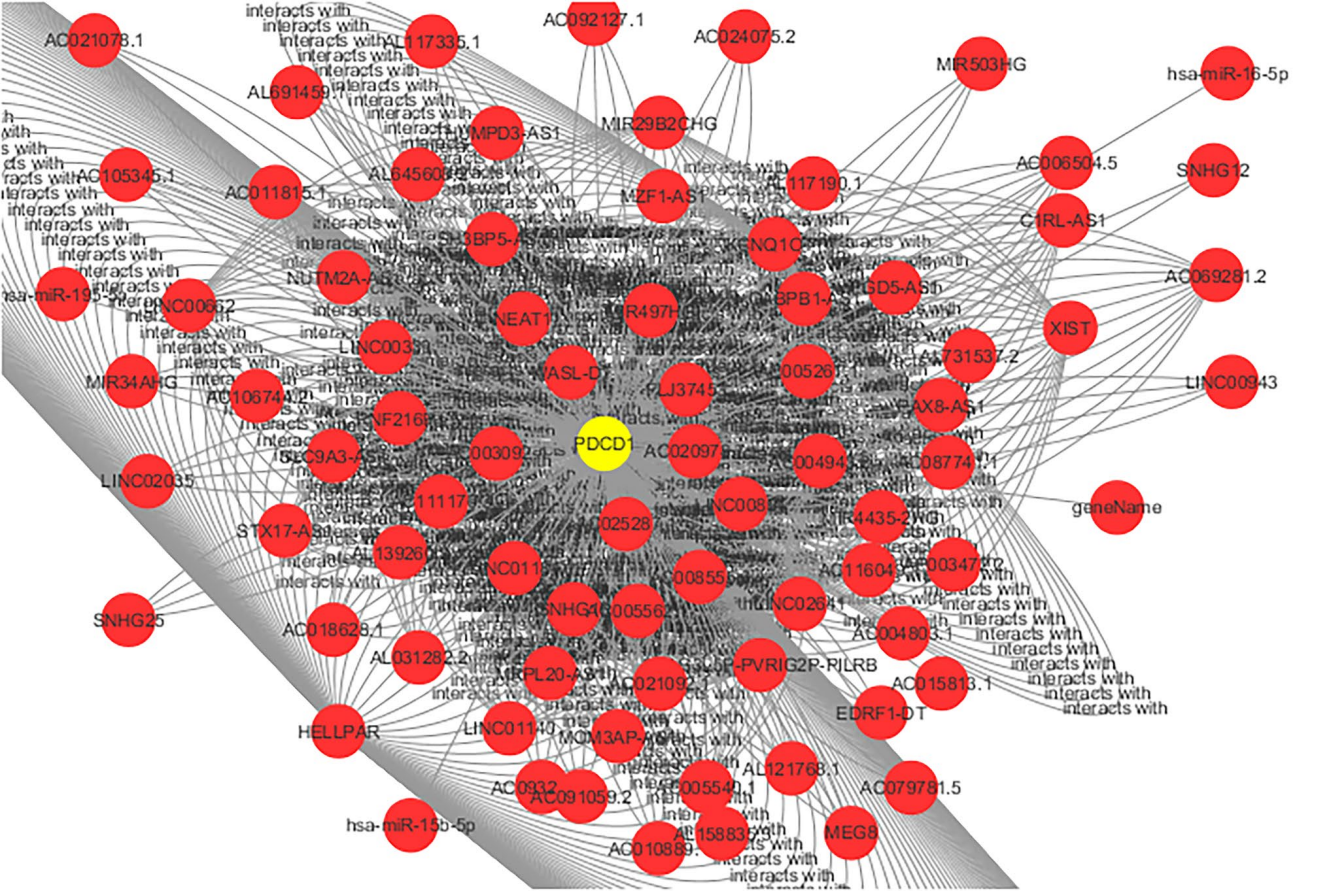
The TF–PDCD1 regulatory network was constructed using Cytoscape, revealing transcriptional regulators potentially involved in the development of ibrutinib resistance

The recognition of PDCD1 as a shared hub gene in two separate datasets (GSE249956 and GSE98206), along with its potential for drug targeting, indicates that immune checkpoint–mediated immune evasion might play a role in ibrutinib resistance in chronic lymphocytic leukemia (CLL). Therefore, targeting PDCD1 with established checkpoint inhibitors could offer a promising alternative or complementary strategy for cases resistant to ibrutinib (Figs. 4 and 5).

## Discussion

In this study, we conducted extensive in silico research to identify key hub genes and regulatory pathways associated with ibrutinib resistance in chronic lymphocytic leukemia (CLL). We identified differentially expressed genes (DEGs) and constructed robust protein–protein interaction (PPI) networks by combining two separate microarray datasets (GSE249956 and GSE98206). This finding enabled us to identify important hub genes that may significantly influence the advancement of the disease and its response to treatment.

Our research identified that PDCD1, CD1C, and ITGB2 are key hub genes consistently found across multiple datasets. This indicates they may play a role in ibrutinib resistance. PDCD1, which codes for the immunotherapeutic target PD-1 molecule, was identified as the strongest candidate because its role was known in the avoidance by tumors of the host’s immunity [20]. Research in the past has established that the overexpression of PDCD1 leads to the persistence and progression of CLL through the suppression of T-cells in the tumor microenvironment [21]. These discoveries confirm this established function as well as engage PDCD1 in acquired resistance against the BTK inhibitors alongside suppression of immunity.

The lncRNA–miRNA–mRNA interaction network suggests a complex post-transcriptional regulation of PDCD1 [22]. The found non-coding RNAs will supposedly function as competitive endogenous RNAs (ceRNAs) that can regulate PDCD1 expression levels and thus potentially influence the effectiveness of ibrutinib [23]. These results offer further evidence that lncRNAs, as well as microRNAs, can be significant regulators of gene expression networks and potentially hold a focal role in the disease process in CLL as a result of drug resistance [24]. In addition, our TF regulatory network indicates that several TFs will potentially target PDCD1 mRNA by modulating its gene expression in CLL [25]. This adds another layer of potential regulation that could be targeted in therapy. The combined ceRNA and TF network models offer a hypothetical view of how multiple regulatory axes might converge on PDCD1 to influence the immune system in CLL [26].

The study of drug–gene interactions revealed that ibrutinib does not directly target PDCD1 but that the PD-1 pathway can be targeted by several FDA-approved immune checkpoint inhibitors, including durvalumab, nivolumab, pembrolizumab, and dostarlimab. These drugs, which inhibit the PD-1/PD-L1 pathway and enhance T-cell activation, are already used in various cancers**.** This enhances T-cell activation against tumor cells. Thus, our discovery points toward combining ibrutinib with PD-1 inhibitors being an approach that ought to be pursued in aiming to counteract prospective immunologic escape as well as resistance patterns in CLL patients unresponsive to monotherapy with BTK inhibitors [27].

Overall, our results align with previous studies showing that ibrutinib targets B-cell receptor signaling and also modifies the tumor microenvironment by influencing immunological checkpoints such as PD-1/PD-L1. As a result, the discovery that PDCD1 is a common hub gene with druggable potential provides a rationale for clinical studies exploring combination therapies that include both BTK inhibitors and immune checkpoint inhibitors in CLL.

This study has several important limitations that must be considered. First, our analysis is computational, and the inferences rely solely on bioinformatics. Therefore**,** to confirm the molecular function of PDCD1 and the regulatory network involved in ibrutinib resistance, future work must include functional assays, and patient-derived samples must be used in experiments. Second, although using multiple independent datasets increases the stability of the identified hub genes, the generalizability of these biomarkers could be further strengthened with larger sample sizes and validation across more diverse cohorts. Lastly, our model is founded on transcriptomics data; incorporating further multi-omics data sources (e.g., proteomics as well as single-cell gene sequencing) would be required to more precisely fine-tune these drug targets as well as comprehend the pertinent biology. Following the comprehensive recent analysis by Maher et al. [28], which clarified the epigenetic landscape of mature B-cell neoplasms, including CLL, we have identified a critical need to integrate these established epigenetic mechanisms with our novel discoveries. Our study delineates regulatory networks and hub genes, notably PDCD1, associated with ibrutinib resistance. Given the recognized importance of epigenetic modifications in ibrutinib therapy, future research could explicitly integrate the findings from Maher et al. concerning DNA methylation, histone modifications, and chromatin remodeling with our transcriptional and post-transcriptional data. This type of integrated approach may provide a more comprehensive understanding of resistance and help identify new therapeutic targets. In brief, our investigation distinguishes PDCD1 as a central hub gene in ibrutinib-resistant CLL and defines the transcription factors as well as non-coding RNAs that govern it. To potentially overcome such resistance and achieve enhanced patient responses, our investigations facilitate exploration on PD-1/PD-L1 inhibitors as an add-on approach to conventional CLL therapies as a groundwork for subsequent experimental investigations.

## Supplementary Information

Below is the link to the electronic supplementary material.

Supplementary file1 (DOCX 16 KB)

## Data Availability

Data is available upon request from the author.
