# Supplementary material for: PDCD1 as a targetable immune checkpoint hub: therapeutic insights for ibrutinib-resistant CLL management
Source: Clin Exp Med. 2025 Nov 18;26(1):19. doi: 10.1007/s10238-025-01942-2 (PMC12628378; doi:10.1007/s10238-025-01942-2)
Supplement: Supplementary file 1 — Supplementary file1 (DOCX 16 KB) [file 10238_2025_1942_MOESM1_ESM.docx]

**Supplementary Table 1.**

| SAMHD1, ACP5, CD68, ANPEP, ORM2, GRN, CD1C, CD27, ITGAX, RGS11, SMAD7, RXRA, FBN1, ANXA4, MSTN, S100A4, SMAD3, BACH2, RYR1, CD40LG, LMNA, LYZ, NT5M, ACTG1, LY96, ANGPT2, PDCD1, JUP, ITGB7, CXCR3, PNOC, PDE6G, RGS16, TCF7L1, ORM1, ITGB2, LDB3, FOXO1, GNA11, IL2RB, TF, CST7, CPNE5, ZBTB16, RAC2, IFITM1, TLN2. |
| --- |

**Supplementary Table 2.** 47 Hub genes from the PPI network of differentially expressed genes (number of nodes: 47, clustering coefficient: 0.404, network centralization: 0.238)

| ACKR2, GZMB, CCR2, SPIB, ALDOC, PFKFB4, HK2, TALDO1, ANG, RNASE4, OSM, TREM1, CLEC10A, TNFRSF9, NR4A1, HSPA1A, HSPA1B, PDCD1, FASLG, CD1D, CD1C, ITGB2, TLR4, XCL2, PAX5, EGR2, CDK2AP2, MANF, COL1A2, HSPA5, DMRTC1, DMRTC1B, DOCK3,NEDD9, DUSP2, EGR1, EGR3, HMOX1, LMO2, TRIB1, H2BC21, EID3, TXNRD1, FCRL5, VPREB3, PIGR, SIGLEC5, FTL, GCLM, TXN, SRXN1, H4C9, HELZ2, OAS3, RSAD2, HHLA2, MAP1A, ICAM2, NKD1,WNT6, RIN2, SH2B2, SORBS1, SIGLEC14, TMC6, TMC8. |
| --- |

**Supplementary Table 3.** 66 Hub genes from the PPI network of differentially expressed genes (number of nodes: 66, clustering coefficient: 0.372, network centralization: 0.200)
